# Impact of Baseline Hypoalbuminemia on Long-Term Survival Following Acute Myocardial Infarction According to Body Mass Index

**DOI:** 10.3390/jcdd11120378

**Published:** 2024-11-26

**Authors:** Alon Shechter, Shani Dahan, Arthur Shiyovich, Harel Gilutz, Ygal Plakht

**Affiliations:** 1 Department of Cardiology, Smidt Heart Institute, Cedars-Sinai Medical Center, Los Angeles, CA 90048, USA; alonshechter@gmail.com; 2 Department of Cardiology, Rabin Medical Center, Petach Tikva 4941492, Israel; arthur.shiyovich@gmail.com; 3 Faculty of Medical and Health Sciences, Tel Aviv University, Tel Aviv 6997801, Israel; 4 Division of Cardiology, Massachusetts General Hospital, Boston, MA 02114, USA; shish.dahan@gmail.com; 5 Department of Cardiology, Assuta Medical Center, Ashdod 7747629, Israel; 6 Goldman Medical School, Faculty of Health Sciences, Ben-Gurion University of the Negev, Beer Sheva 8410501, Israel; gilutz@bgu.ac.il; 7 Cardiovascular Division, Department of Medicine, Harvard Medical School, Boston, MA 02115, USA; 8 Department of Nursing, Recanati School for Community Health Professions, Faculty of Health Sciences, Ben-Gurion University of the Negev, Beer Sheva 84105, Israel; 9 Department of Emergency Medicine, Soroka University Medical Center, Beer Sheva P.O. Box 151, Israel

**Keywords:** albumin, body mass index, myocardial infarction, survival

## Abstract

Serum albumin and body mass index (BMI, kg/m^2^) have been associated with outcomes following acute myocardial infarction (AMI). Aiming to assess whether the mortality risk inflicted by hypoalbuminemia (<3.5 g/dL) in this context is influenced by BMI, we conducted a retrospective analysis of AMI survivors hospitalized during 2004–2017. Stratified by admission-time albumin level and BMI, eligible cases were evaluated for all-cause mortality up to 10 years after discharge. A total of 6283 individuals (74.1% males, mean age 64.1 ± 13.1 years, 44.3% with ST-elevation MI) were included. Of them, 22.7% had hypoalbuminemia and 1.2%, 41.0%, and 28.6% were underweight (BMI < 18.5), overweight (BMI 25–30), and obese (BMI ≥ 30), respectively. Over a median of 7.9 (IQR, 4.8–10.0) years of follow-up, 42.5% of patients died. Hypoalbuminemia was independently associated with a heightened mortality risk overall (AdjHR = 1.54, 95%CI 1.42–1.67, *p* < 0.001), accounted for by the normal weight (AdjHR = 1.73, 95%CI 1.50–1.99, *p* < 0.001), overweight (AdjHR = 1.55, 95%CI 1.35–1.79, *p* < 0.001), and class 1 obesity (BMI 30–35) (AdjHR = 1.37, 95%CI 1.12–1.68, *p* = 0.002) subgroups. Upon interaction analysis, the mortality risk imposed by hypoalbuminemia was most pronounced among individuals with normal BMI. In conclusion, hypoalbuminemia constituted a negative prognostic marker for long-term survival in AMI patients with normal or mildly elevated but not reduced or severely increased BMI. Pending further research, addressing hypoalbuminemia based on BMI range may prove beneficial.

## 1. Introduction

Over the last decades, significant improvement in survival after acute myocardial infarction (AMI) has led to an extensive search for prognostic indicators to further enhance disease management and outcomes. Among the numerous factors explored, serum albumin and body mass index (BMI) have emerged as particularly intriguing variables with potential impact on the post-MI course [1,2]. Albumin, a vital component of plasma, which plays a multifaceted role in various physiological processes [3], has been shown to mark nutritional status, inflammation extent, organ (e.g., kidneys, liver) function, and numerous illnesses severity [4]. Accordingly, low serum albumin levels have been linked to a higher risk of incident AMI and chronic coronary artery disease (CAD) [5,6]. Furthermore, among AMI patients, decreased serum albumin upon admission has been associated with worse in-hospital outcomes (death included) and long-term survival as well as no-reflow in those with ST-elevation MI who had undergone primary percutaneous intervention [7,8,9]. BMI, an anthropometric measure representing the ratio of weight (in kg) to height (in m squared) [10], has demonstrated contradicting implications in cardiac patients—collectively referred to as the “obesity paradox”—in which higher values denote elevated risk of cardiovascular morbidity but also improved prognosis among patients with the established disease [11,12,13,14,15]. Acknowledging the possible modifying effect of BMI on outcomes, we aimed to assess whether the prognostic implication of hypoalbuminemia among AMI survivors is indeed altered by BMI.

## 2. Materials and Methods

### 2.1. Study Population and Outcomes

Our study represents a retrospective analysis of the Soroka University Medical Center (SUMC) registry of consecutive AMI hospitalizations occurring between 1 June 2004 and 31 October 2017. Included in the study were adult (i.e., ≥18-year-old) Israeli citizens who were hospitalized with AMI as the primary/main diagnosis and survived the index event and for whom there were available data regarding serum albumin level and BMI at admission. For subjects with multiple AMI admissions at SUMC, only the first one was considered. The study outcome was all-cause mortality up to 10 years after hospital discharge or 31 July 2023, whichever occurred first.

This project conformed to the Declaration of Helsinki and was approved by Soroka’s Institutional Review Board (approval number SOR-0319-16), which waived the need for informed consent in view of the investigation’s retrospective nature.

### 2.2. Data Collection and Definitions

Clinical data were retrieved from a web-based medical chart platform, in which baseline comorbidities were identified by the International Classification of Diseases, Ninth Revision, Clinical Modification (ICD-9-CM) [16] codes, as documented in real time by the treating medical team and according to the prespecified criteria outlined below. Death events were obtained from the Israeli Ministry of the Interior Population Registry.

AMI diagnosis was based on the constellation of ischemic signs and/or symptoms coupled with an abrupt rise and fall in cardiac biomarkers levels consistent with acute myocardial injury, as dictated by the Universal Definition of Myocardial Infarction at the time [17]. Obstructive CAD required the presence of a ≥70% vessel stenosis, as assessed by angiography.

Hypoalbuminemia was defined as a serum albumin level of <3.5 g/dL [18]. BMI classification followed the Centers for Disease Control and Prevention’s scheme [19], resulting in six distinct categories: underweight—<18.5 kg/m^2^; normal weight—18.5 to <25 kg/m^2^; overweight—25 to <30 kg/m^2^; class 1 obesity—30 to <35 kg/m^2^; class 2 obesity—35 to <40 kg/m^2^; and class 3 (or morbid) obesity—≥40 kg/m^2^. Importantly, only the first measurements reported within the initial 24 h of admission were taken into consideration. The presence of diabetes mellitus and dyslipidemia was ascertained by HbA1c and low-density lipoprotein levels of ≥6.5% and ≥100 mg/dL, respectively, at any timepoint during a 12-month period starting 6 months before the hospitalization.

Echocardiographic diagnoses followed the American Society of Echocardiography guidelines. Specifically, severe left ventricular (LV) dysfunction was defined by an LV ejection fraction (LVEF) of <30% on the first in-hospital echocardiogram. Pulmonary hypertension was declared upon a pulmonary arterial systolic pressure of ≥37 mmHg on the same exam.

### 2.3. Statistical Analysis

The study cohort was analyzed in its entirety and according to hypoalbuminemia status and BMI category at admission. Variables were reported as frequencies and percentages, medians and interquartile ranges (IQRs), or means and standard deviations, and compared using Pearson’s Chi-Square, Fisher’s exact, Student’s *t*, and analysis of variance (ANOVA) tests. Predictors for hypoalbuminemia in the total cohort were identified using a binary logistic regression multivariable analysis, which incorporated baseline variables demonstrating a *p*-value of <0.1 at the univariate stage.

The time-dependent probability and cumulative incidence of mortality as a function of hypoalbuminemia and various BMI ranges were assessed by the Kaplan–Meier method and compared using the Log-Rank test. Independent associations with the risk for all-cause death, both in the total cohort and in each of the BMI categories subgroups, were evaluated by a Cox proportional hazard multivariable analysis, using a stepwise approach as described above. Lastly, an interaction analysis was undertaken to determine the relative prognostic value of hypoalbuminemia in relation to a BMI range other than normal.

Cases with missing values were censored from the relevant calculations. Statistical significance required a two-sided *p*-value of <0.05. All analyses were performed using Statistical Package for the Social Sciences (SPSS), version 29 (IBM Corporation, Armonk, NY, USA).

## 3. Results

### 3.1. Baseline Characteristics of the Study Population

Out of 15,329 AMI hospitalizations identified at SUMC between 2004 and 2017, 6283 (41.0%) were first-time admissions of Israeli citizens who survived to discharge and had documented albumin and BMI values during the first 24 h of hospital stay (Supplemental Figure S1). Among these, hypoalbuminemia was observed in 1425 (22.7%) (Table 1). Concurrently, underweight was diagnosed in 77 (1.2%) subjects, normal weight in 1825 (29.1%), overweight in 2578 (41.0%), class 1 obesity in 1303 (20.7%), class 2 obesity in 372 (5.9%), and class 3 obesity in 128 (2.0%). Overall, the serum albumin level increased and the prevalence of hypoalbuminemia decreased as the BMI was higher—from 50.6% in underweight patients to 23.4% in those with class 3 obesity (*p* for trend < 0.001) (Supplemental Figure S2 and Table S1). BMI, on its part, was lower among hypoalbuminemic compared to normoalbuminemic patients, at 26.9 ± 5.3 vs. 28.1 ± 4.8 kg/m^2^ (*p* < 0.001). Notably, higher odds for hypoalbuminemia were associated with older age, female sex, non-Jewish minority, comorbidities (other than overweight/obesity and dyslipidemia), lack of prior revascularization, and an ST-elevation MI presentation (Supplemental Table S2).

Both patients with hypoalbuminemia and those exhibiting a lower BMI were older and more likely to be male and to exhibit a greater burden of non-CV comorbidities compared to non-hypoalbuminemic and higher-range BMI patients, respectively (Table 1 and Supplemental Table S1). By contrast, the CV risk factors and comorbidities distribution was more heterogenous.

The trends associated with hypoalbuminemia in the total cohort were largely maintained within the normal to moderately elevated BMI categories (i.e., normal weight to class 2 obesity) subgroups. Among patients at BMI extremes (i.e., with underweight or class 3 obesity), however, CV morbidity—rather than general demographics and non-CV conditions—was related to serum albumin status, demonstrating a higher frequency in hypoalbuminemic individuals (Supplemental Tables S3 and S4).

### 3.2. Acute Event Aspects

Mirroring baseline characteristics, patients with vs. those without hypoalbuminemia and those with a lower BMI presented more often with cardiogenic shock and non-ST-elevation MI (Table 2 and Supplemental Table S5). Such patients were also more likely to display severe LV dysfunction, pulmonary hypertension, and significant valvular regurgitation upon admission, and their coronary angiograms revealed a greater extent of obstructive CAD. Notably, echocardiographic and angiographic assessment as well as revascularization therapy were employed less frequently among subjects with hypoalbuminemia and lower BMI. The hospitalization course in these patients was lengthier and more complex, involving higher rates of sepsis, mechanical ventilation, and blood transfusion.

Once again, most findings associated with hypoalbuminemia were observed only among patients with either normal weight, overweight, or class 1–2 obesity (Supplemental Tables S6 and S7). Accordingly, within the underweight and class 3 obesity subgroups, patients with normal and low serum albumin experienced a more comparable course.

### 3.3. Outcome

By a median of 7.9 (IQR, 4.8–10.0) years of follow-up, a total of 2669 (42.5%) patients died (Supplemental Table S8). The mortality rates were higher in the presence of hypoalbuminemia (66.2%) and BMI extremes (77.9% and 53.9% among underweight and class 3 obese patients, respectively)—compared to normoalbuminemia (35.5%) and normal-range BMI (47.5%) (all *p* < 0.001). Likewise, cumulative survival was reduced in the low vs. normal serum albumin and lower BMI subgroups (Supplemental Figure S3).

**Figure 1:**
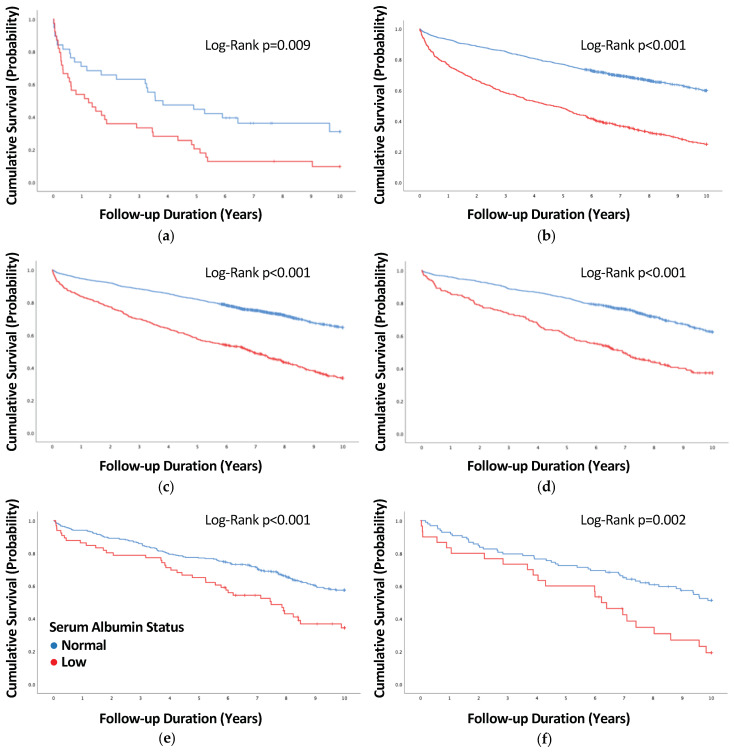
Cumulative survival according to serum albumin status and body mass index category: (**a**) underweight; (**b**) normal weight; (**c**) overweight; (**d**) class 1 obesity; (**e**) class 2 obesity; (**f**) class 3 obesity. BMI = body mass index.

The increased rate and cumulative incidence of mortality associated with hypoalbuminemia in the total cohort were also observed in each of the BMI categories subgroups. Concurrently, the univariate (i.e., unadjusted) hazard ratios imposed by hypoalbuminemia demonstrated a somewhat U-shape trend, reaching a peak within the normal to mildly elevated BMI (i.e., normal weight to class 1 obesity) subgroups. The excess mortality linked to BMI extremes was evident regardless of hypoalbuminemia status (Supplemental Table S8 and Figure 1).

Per multivariable analysis, both decreasing albumin level (as a continuous variable), hypoalbuminemia (vs. normoalbuminemia), and below-normal (vs. normal) BMI independently conferred a higher 10-year mortality risk (AdjHR for 1 g/dL-decrease 1.61, 95% CI 1.48–1.75, AdjHR 1.54, 95% CI 1.42–1.67, and AdjHR 1.98, 95% CI 1.52–2.58, respectively, all *p* < 0.001) (Table 3). Notably, there was a steep rise in the risk at a serum albumin level of around 3.5 g/dL, corresponding to the cutoff level used to define hypoalbuminemia (Supplemental Figure S4). Contrasting the afore-mentioned, an above-normal (compared with normal) BMI was associated with either a reduced risk (overweight to class 2 obesity) (AdjHR 0.74 to 0.83, *p* < 0.001 to 0.037) or a similar risk (class 3 obesity) (AdjHR 1.10, 95% CI 0.86–1.41, *p* = 0.464).

**Figure 2:**
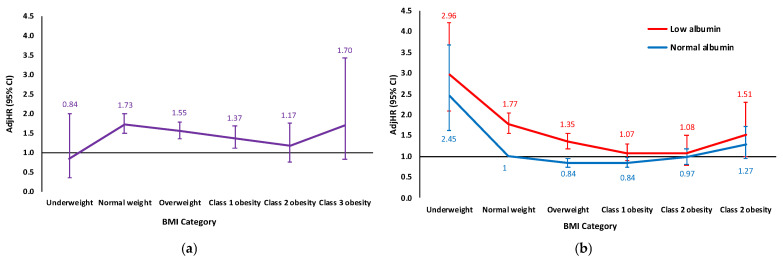
Hypoalbuminemia-associated risk of all-cause mortality at 10 years after acute myocardial infarction according to body mass index category: (**a**) per multivariable analysis in each of the BMI categories’ subgroups; (**b**) per interaction analysis in the total cohort. AdjHR = adjusted hazard ratio; BMI = body mass index; CI = confidence interval.

The hazard imposed by low serum albumin status was only manifested in patients presenting at hospitalization with either a normal BMI (AdjHR 1.73, 95% CI 1.50–1.99, *p* < 0.001), overweight (AdjHR 1.55, 95% CI 1.35–1.79, *p* < 0.001), or class 1 obesity (AdjHR 1.37, 95% CI 1.12–1.68, *p* = 0.002)—but not with underweight (AdjHR 0.84, 95% CI 0.35–2.00, *p* = 0.698), class 2 obesity (AdjHR 1.17, 95% CI 0.77–1.75, *p* = 0.465), or class 3 obesity (AdjHR 1.70, 95% CI 0.84–3.42, *p* = 0.137) (Supplemental Tables S9 and S10, and Figure 2). Likewise, a 1 g/dL decrease in serum albumin level independently conferred an increased mortality risk only within the normal weight to class 1 obesity subgroups (Supplemental Figure S5). Upon interaction analysis, hypoalbuminemia demonstrated a weaker association with mortality among patients with abnormal vs. normal BMI, reaching statistical significance in the class 1–2 obesity subgroups (Table 3 and Figure 2).

## 4. Discussion

Our study examined the long-term mortality effect of admission-time hypoalbuminemia, defined as a serum albumin level of <3.5 g/dL, according to BMI range among 6283 AMI survivors. Its main findings were as follows: 1. averaging 22.7% in the total cohort, the prevalence of hypoalbuminemia was overall inversely proportional to BMI value and highest at 50.6% (or lowest at 24.3%) among underweight (or morbidly obese) subjects; 2. compared to normal albumin, hypoalbuminemia was accompanied by a greater burden of comorbidities at baseline, a more complex presentation and hospitalization course and, ultimately, a reduced 10-year survival following the index event; however this was only evident within the normal weight to class 2 obesity (i.e., 18.5 to <40 kg/m^2^) subgroups; and 3. the independent association between hypoalbuminemia and increased risk for mortality was confined to patients with normal to mildly increased BMI (i.e., 18.5 to <35 kg/m^2^), and the hazardous impact of hypoalbuminemia was generally most pronounced in normal weight individuals.

To our knowledge, the current study is the first to report on the interplay between albumin and BMI in the setting of AMI. Stemming from a relatively large registry that incorporated patient-level data and a long follow-up period, the study’s observations are supported by comprehensive regression models, all reinforcing the validity and applicability to the constantly growing population of AMI survivors. Taken together, and in view of the concomitantly expanding obesity pandemic, we believe the study is timely and addresses an important and widely relevant topic in the acute coronary syndrome arena that may carry actionable implications, as outlined below.

Two main ‘take-home’ messages may be offered by our study. The first is that a higher-risk profile and more adverse outcomes that classically accompany hypoalbuminemia may not apply to underweight and moderately to extremely obese individuals recovering from AMI. Despite the small representation of these BMI subgroups in the study’s cohort (n = 577, 9.2%), our results arguably suggest a genuine modifying effect of BMI on the prognostic implications of hypoalbuminemia nonetheless, as the mortality rate was nominally highest within the aforementioned subgroups as well. One possible explanation relating to the lower BMI extreme is that a higher comorbid state (and, presumably, malnutrition) associated with underweight may have dictated a less favorable outcome irrespective of albumin level. As for the above-normal BMI extreme, hypoalbuminemia may have been less reflective of poor nutrition and any associated phenomena (e.g., energy crisis, infections) in patients with pronounced obesity. Alternatively, it could be that a greater endothelial dysfunction, previously shown to characterize obese patients [20], played a more decisive prognostic role, somewhat overshadowing the effect of hypoalbuminemia. Still further, one may speculate about the well-documented and poorly understood obesity paradox that led to hypoalbuminemia being less prognostically meaningful in patients with obesity. As suggested by prior works [21], it could be that secondary preventive measures, not explicitly explored in our database, were more extensive among obese subjects, thus counteracting the deleterious effects of hypoalbuminemia. Due to the study’s retrospective nature and lack of information on death causes and hypoalbuminemia etiologies, we were not able to determine causality and reliably identify the mechanisms underlying our results. Accordingly, these may best be attested by future prospective research.

The second, more practical notion arising from our study is that consideration of both albumin status and BMI range, as opposed albumin status alone, could allow for a more personalized risk stratification both before and after AMI, which in turn may translate to a more targeted management and surveillance scheme and, potentially, improved resource utilization and patient outcome. Theoretically, as hypoalbuminemia demonstrated an independent predictive capacity for death among AMI patients with normal weight to mild obesity only, it seems rational to focus diagnostic, preventive, and corrective measures of a low serum albumin level (e.g., dietary consultation, nutritional support, and albumin replacement) in this subset of individuals. In the same sense, the presence of hypoalbuminemia in AMI patients at BMI extremes may be regarded as less prognostically alarming on its own, thereby emphasizing the importance of addressing these BMI deviations while sparing the potential cost and hazards (e.g., allergic reactions, volume overload, and infections) associated with (parenteral) hypoalbuminemia treatment, the efficacy of which has yet to be established in CV patients, particularly in terms of primary and secondary prevention of ACS [22]. This implication of specifically targeting normal weight to mildly obese subjects—rather than moderately to severely obese ones—may apply to primary prevention pathways (not assessed in the current study) as well and provide an interesting perspective to practice guidelines [23], which at present mainly focus on the more obese patients. Once again, further, preferentially multi-center, studies are needed to evaluate the above-mentioned hypotheses and their application in actual clinical practice.

### Limitations

First, our study is the product of a single-center, retrospective analysis that did not employ external adjudication and in which more than half of patients were excluded (mostly due to the absence of data regarding baseline BMI), all of which could lead to selection bias and hamper the generalizability of the results. However, we relied on one of the largest cohorts reported thus far, which represents a population of close to 1 million and whose baseline characteristics resembled those of prior real-world publications. Secondly, some complications (e.g., sepsis) were rather uncommon, thereby altering statistical power and possibly the final multivariable models. Thirdly, the protracted timeframe of enrollment may have led to inconsistencies in medical definitions and treatment approaches, which could have affected the interpretation of the findings. Nevertheless, all patients were exposed to similarly evolving diagnostic criteria and practices. Fourthly, hypoalbuminemia was mainly analyzed as a dichotomous variable, using a cutoff value that has been defined according to a steady-state scenario. While potentially introducing misclassification bias by disregarding the fluctuating nature of serum albumin level, we believe this approach a. is more practical in an acute setting and b. may possess greater clinical relevance compared to a continuous variable-centered analysis, the limited results of which further suggested the 3.5 g/dL cutoff used to define hypoalbuminemia as the most significant prognostically. Fifthly, we were not aware of hypoalbuminemia and abnormal BMI causes (e.g., metabolic, inflammatory, nutritional, pharmacologic, etc.) and durations, which by themselves could affect outcomes. Similarly, our database did not include information on markers of inflammation, medical therapies (other than blood transfusion), or specific measures of hemodynamic status and MI type (most importantly type 2 MI). Consequently, we could not determine the pathophysiologic basis accounting for our observations or rule out confounding by non-coronary-related phenomena. These setbacks were addressed by a. deliberately excluding patients whose primary admission diagnosis was not AMI (e.g., acute illness, infection, etc.) and b. performing extensive multivariable analyses that controlled for baseline and acute event characteristics as well as hospitalization parameters. Lastly, we did not have data on cardiac biomarkers levels. Yet, we believe this knowledge gap did not alter the study’s findings, as a. such levels are highly dependent upon testing timing, accompanying disease states (e.g., renal failure), and the kits used, all of which could differ substantially between patients, and b. we did consider paralleling aspects of MI severity, including the presence of severe LV dysfunction, the number of diseased vessels on angiography, and cardiogenic shock presentation.

## 5. Conclusions

In our large single-center experience, hypoalbuminemia constituted an adverse prognostic marker for long-term survival in AMI patients with normal or mildly elevated—but not with reduced or severely increased—BMI. Pending future research, tailoring the management of hypoalbuminemia in these cases based on BMI range may prove beneficial.

## Figures and Tables

**Table 1:** Baseline clinical characteristics according to serum albumin status.

	Total Cohort (n = 6283)	Serum Albumin Status		*p*-Value *
		Normal (n = 4858)	Low (n = 1425)	
**Demographic Details**				
Age (years)	64.1 ± 13.1	62.8 ± 13.0	68.6 ± 12.5	**<0.001** †
Sex Male	4657 (74.1)	3753 (77.3)	904 (63.4)	**<0.001**
Non-Jewish Minority	1123 (17.9)	842 (17.3)	281 (19.7)	**<0.001**
**Cardiovascular Risk Factors**				
Diabetes Mellitus	2782 (44.3)	2040 (42.0)	742 (52.1)	**<0.001**
Dyslipidemia	5304 (84.4)	4202 (86.5)	1102 (77.3)	**<0.001**
Hypertension	3477 (55.3)	2683 (55.2)	794 (55.7)	0.743
Smoking History	2999 (47.7)	2446 (50.3)	553 (38.8)	**<0.001**
Family History of IHD	725 (11.5)	627 (12.9)	98 (6.9)	**<0.001**
**Cardiovascular Morbidity**				
Ischemic Heart Disease	5476 (87.2)	4328 (89.1)	1148 (80.6)	**<0.001**
History of MI	989 (15.7)	739 (15.2)	250 (17.5)	**0.034**
Prior Revascularization				
PCI	1102 (17.5)	869 (17.9)	233 (16.4)	0.180
CABG	592 (9.4)	456 (9.4)	136 (9.5)	0.858
Peripheral Arterial Disease	767 (12.2)	518 (10.7)	249 (17.5)	**<0.001**
Atrial Fibrillation/Flutter	958 (15.2)	642 (13.2)	316 (22.2)	**<0.001**
Atrioventricular Block	259 (4.1)	181 (3.7)	78 (5.5)	**0.004**
Clinical Heart Failure	1040 (16.6)	657 (13.5)	383 (26.9)	**<0.001**
**Non-Cardiovascular Morbidity**				
COPD	533 (8.5)	355 (7.3)	178 (12.5)	**<0.001**
Stage ≥ III CKD	577 (9.2)	322 (6.6)	255 (17.9)	**<0.001**
Anemia	3043 (48.4)	2066 (42.5)	977 (68.6)	**<0.001**
Neurological Disorders	949 (15.1)	624 (12.8)	325 (22.8)	**<0.001**
Malignancy	603 (9.8)	412 (8.5)	191 (13.4)	**<0.001**
Psychotic Disorders	86 (1.4)	56 (1.2)	30 (2.1)	**0.007**
Alcohol/Drug Abuse	140 (2.2)	108 (2.2)	32 (2.2)	0.960
**Serum Albumin**				
Mean Level (g/dL)	3.7 ± 0.5	3.9 ± 0.3	3.1 ± 0.3	**<0.001** †
**Body Mass Index**				
Mean Value (kg/m^2^)	27.8 ± 5.0	28.1 ± 4.8	26.9 ± 5.3	**<0.001** †
Category				**<0.001**
Underweight	77 (1.2)	38 (0.8)	39 (2.7)	
Normal Weight	1825 (29.1)	1296 (26.7)	529 (37.1)	
Overweight	2578 (41.0)	2073 (42.7)	505 (35.4)	
Class 1 Obesity	1303 (20.7)	1047 (21.6)	256 (18.0)	
Class 2 Obesity	372 (5.9)	306 (6.3)	66 (4.6)	
Class 3 Obesity	128 (2.0)	98 (2.0)	30 (2.1)	

Data are presented as number (percent) or mean ± standard deviation. Figures in bold denote statistical significance. * Chi-square test unless stated otherwise; † Student’s *t*-test. CABG = coronary artery bypass grafting; CKD = chronic kidney disease; COPD = chronic obstructive pulmonary disease; IHD = ischemic heart disease; MI = myocardial infarction; PCI = percutaneous coronary intervention.

**Table 2:** Acute event aspects according to serum albumin status.

	Total Cohort (n = 6283)	Serum Albumin Status		*p*-Value *
		Normal (n = 4858)	Low (n = 1425)	
**Clinical Presentation**				
Cardiac Arrest	28 (0.4)	15 (0.3)	13 (0.9)	**0.003**
Cardiogenic Shock	106 (1.7)	48 (1.0)	58 (4.1)	**<0.001**
ST-Elevation MI	2784 (44.3)	2187 (45.0)	597 (41.9)	**0.037**
**Echocardiographic Parameters**				
Echocardiogram Performed	5186 (82.5)	4094 (84.3)	1092 (76.6)	**<0.001**
Severe LV Dysfunction	620 (12.0)	396 (9.7)	224 (20.5)	**<0.001**
LV Hypertrophy	300 (5.8)	224 (5.5)	76 (7.0)	0.061
Mitral Regurgitation	319 (6.2)	196 (4.8)	123 (11.3)	**<0.001**
Tricuspid Regurgitation	189 (3.6)	113 (2.8)	76 (7.0)	**<0.001**
Pulmonary Hypertension	398 (7.7)	252 (6.2)	146 (13.4)	**<0.001**
**Angiographic Parameters**				
Angiogram Performed	4793 (76.3)	3880 (79.9)	913 (64.1)	**<0.001**
Vessels Significantly Involved				**<0.001**
0	168 (3.5)	132 (3.4)	36 (3.9)	
1	1106 (23.1)	929 (23.9)	177 (19.4)	
2	1284 (26.8)	1062 (27.4)	222 (24.3)	
3/Left Main	2235 (46.6)	1757 (45.3)	478 (52.4)	
**Hospital Course**				
Revascularization Approach				**<0.001**
No/Conservative Treatment	1063 (16.9)	646 (13.3)	417 (29.3)	
PCI	3737 (59.5)	3047 (62.7)	690 (48.4)	
CABG	1483 (23.6)	1165 (24.0)	318 (22.3)	
Intra-Aortic Balloon Pulsation	172 (2.7)	82 (1.7)	90 (6.3)	**<0.001**
Any Form of Pacing	136 (2.2)	83 (1.7)	53 (3.7)	**<0.001**
Mechanical Ventilation	246 (4.2)	122 (2.5)	142 (10.0)	**<0.001**
Gastrointestinal Bleeding	147 (2.3)	76 (1.6)	71 (5.0)	**<0.001**
Blood Transfusion	1019 (16.2)	649 (13.4)	370 (26.0)	**<0.001**
Sepsis	84 (1.3)	23 (0.5)	61 (4.3)	**<0.001**
Intensive Care Unit Stay	4675 (74.4)	3727 (76.7)	948 (66.5)	**<0.001**
Hospitalization Length (days)	11.3 ± 9.9	10.4 ± 8.5	14.3 ± 13.0	**<0.001** †

Data are presented as number (percent) or mean ± standard deviation, as appropriate. Figures in bold denote statistical significance. * Chi-square test unless stated otherwise; † Student’s *t*-test. CABG = coronary artery bypass grafting; LV = left ventricular; MI = myocardial infarction; PCI = percutaneous coronary intervention.

**Table 3:** Multivariable Cox proportional hazard model for the outcome of all-cause mortality at 10 years and interaction analysis.

Parameter	AdjHR (95% CI)	*p*-Value *
Age (vs. <65 years)		
65–74 years	1.97 (1.77–2.18)	**<0.001**
≥75 years	3.14 (2.83–3.49)	**<0.001**
Diabetes Mellitus	1.38 (1.27–1.50)	**<0.001**
Dyslipidemia	0.91 (0.83–1.01)	0.076
Family History of Ischemic Heart Disease	0.64 (0.52–0.79)	**<0.001**
History of Myocardial Infarction	1.18 (1.07–1.30)	**<0.001**
Peripheral Arterial Disease	1.32 (1.19–1.45)	**<0.001**
Atrial Fibrillation/Flutter	1.37 (1.26–1.51)	**<0.001**
Clinical Heart Failure	1.24 (1.13–1.36)	**<0.001**
Chronic Obstructive Pulmonary Disease	1.65 (1.48–1.85)	**<0.001**
Stage ≥ III Chronic Kidney Disease	1.70 (1.53–1.89)	**<0.001**
Anemia	1.39 (1.27–1.51)	**<0.001**
Neurological Disorders	1.54 (1.40–1.68)	**<0.001**
Malignancy	1.77 (1.53–2.06)	**<0.001**
Alcohol/Drug abuse	1.62 (1.27–2.07)	**<0.001**
Non-ST Elevation vs. ST-Elevation Myocardial Infarction	1.24 (1.14–1.36)	**<0.001**
Severe Left Ventricular Dysfunction	1.44 (1.28–1.62)	**<0.001**
Left Ventricular Hypertrophy	1.37 (1.17–1.61)	**<0.001**
Tricuspid Regurgitation	1.28 (1.07–1.54)	**0.007**
Pulmonary Hypertension	1.27 (1.11–1.45)	**<0.001**
Revascularization Approach (vs. Conservative):		
Percutaneous Coronary Intervention	0.57 (0.52–0.63)	**<0.001**
Coronary Artery Bypass Grafting	0.42 (0.37–0.47)	**<0.001**
**Serum Albumin Status and Body Mass Index Category**		
Decreasing Serum Albumin Level (continuous, per 1 g/dL decrease)	1.61 (1.48–1.75)	**<0.001**
Low vs. Normal Serum Albumin Level	1.54 (1.42–1.67)	**<0.001**
Abnormal vs. Normal Weight		
Underweight	1.98 (1.52–2.58)	**<0.001**
Overweight	0.80 (0.73–0.88)	**<0.001**
Class 1 Obesity	0.74 (0.67–0.83)	**<0.001**
Class 2 Obesity	0.83 (0.70–0.99)	**0.037**
Class 3 Obesity	1.10 (0.86–1.41)	0.464
**Interaction Analysis: Abnormal Weight x Hypoalbuminemia**		
Underweight x Hypoalbuminemia	0.69 (0.40–1.17)	0.167
Overweight x Hypoalbuminemia	0.91 (0.75–1.10)	0.322
Class 1 Obesity x Hypoalbuminemia	0.73 (0.58–0.92)	**0.008**
Class 2 Obesity x Hypoalbuminemia	0.63 (0.43–0.92)	**0.017**
Class 3 Obesity x Hypoalbuminemia	0.67 (0.40–1.14)	0.139

Figures in bold denote statistical significance. * Cox regression analysis. AdjHR = adjusted hazard ratio; CI = confidence interval.

## Data Availability

The data underlying this article will be shared upon reasonable request to the corresponding author.

## Supplementary Materials

The following supporting information can be downloaded at https://www.mdpi.com/article/10.3390/jcdd11120378/s1, Figure S1: Study flow chart; Figure S2: Hypoalbuminemia prevalence and serum albumin level according to body mass index category; Figure S3: Cumulative survival: (a) according to serum albumin status; (b) according to body mass index category; Figure S4: Serum albumin decrease-associated risk of all-cause mortality at 10 years after acute myocardial infarction per multivariable analysis in the total cohort; Figure S5: Serum albumin decrease-associated risk of all-cause mortality at 10 years after acute myocardial infarction per multivariable analysis in each of the body mass index categories subgroups; Table S1: Baseline clinical characteristics according to body mass index category; Table S2: Multivariable binary logistic regression model for admission-time hypoalbuminemia in the total cohort; Table S3: Baseline clinical characteristics of patients with underweight, normal weight, and overweight, stratified by serum albumin status; Table S4: Baseline clinical characteristics of patients with obesity, stratified by serum albumin status; Table S5: Acute event aspects by body mass index category; Table S6: Acute event aspects in patients with underweight, normal weight, and overweight, stratified by serum albumin status; Table S7: Acute event aspects in patients with obesity, stratified by serum albumin status; Table S8: Ten-year all-cause mortality; Table S9: Multivariable Cox proportional hazard models for the outcome of all-cause mortality at 10 years in patients with underweight, normal weight, and overweight; Table S10: Multivariable Cox proportional hazard models for the outcome of all-cause mortality at 10 years in patients with obesity.
