# Supplementary material for: Impact of Baseline Hypoalbuminemia on Long-Term Survival Following Acute Myocardial Infarction According to Body Mass Index
Source: J Cardiovasc Dev Dis. 2024 Nov 26;11(12):378. doi: 10.3390/jcdd11120378 (PMC11676636; doi:10.3390/jcdd11120378)
Supplement: Supplementary file 1 [file jcdd-11-00378-s001.zip › jcdd-3265947-supplementary.pdf]

SUPPLEMENT

Supplemental Figures

Supplemental Figure S1	2
Supplemental Figure S2	3
Supplemental Figure S3	4
Supplemental Figure S4	6
Supplemental Figure S5	7

Supplemental Tables

Supplemental Table S1	8
Supplemental Table S2	10
Supplemental Table S3	11
Supplemental Table S4	13
Supplemental Table S5	15
Supplemental Table S6	17
Supplemental Table S7	19
Supplemental Table S8	21
Supplemental Table S9	22
Supplemental Table S10	24

Supplemental Figure S1. Study Flow Chart

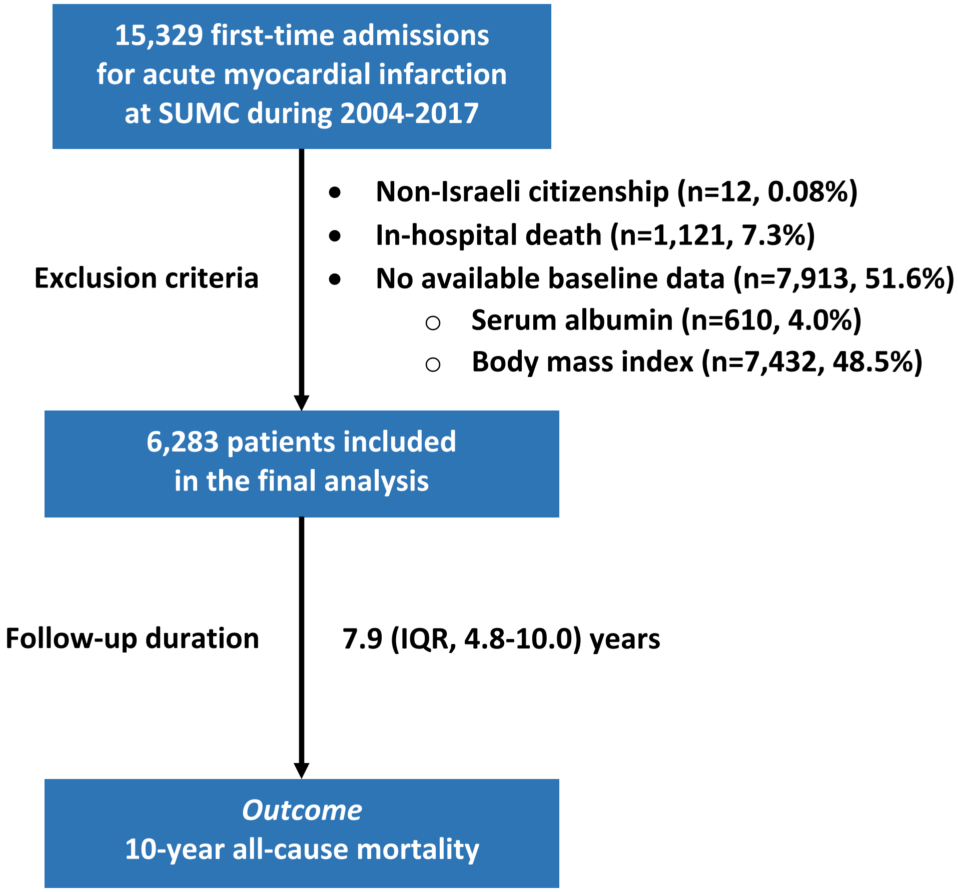

IQR = interquartile range; SUMC = Soroka University Medical Center.

Supplemental Figure S2. Hypoalbuminemia Prevalence and Serum Albumin Level According to Body Mass Index Category

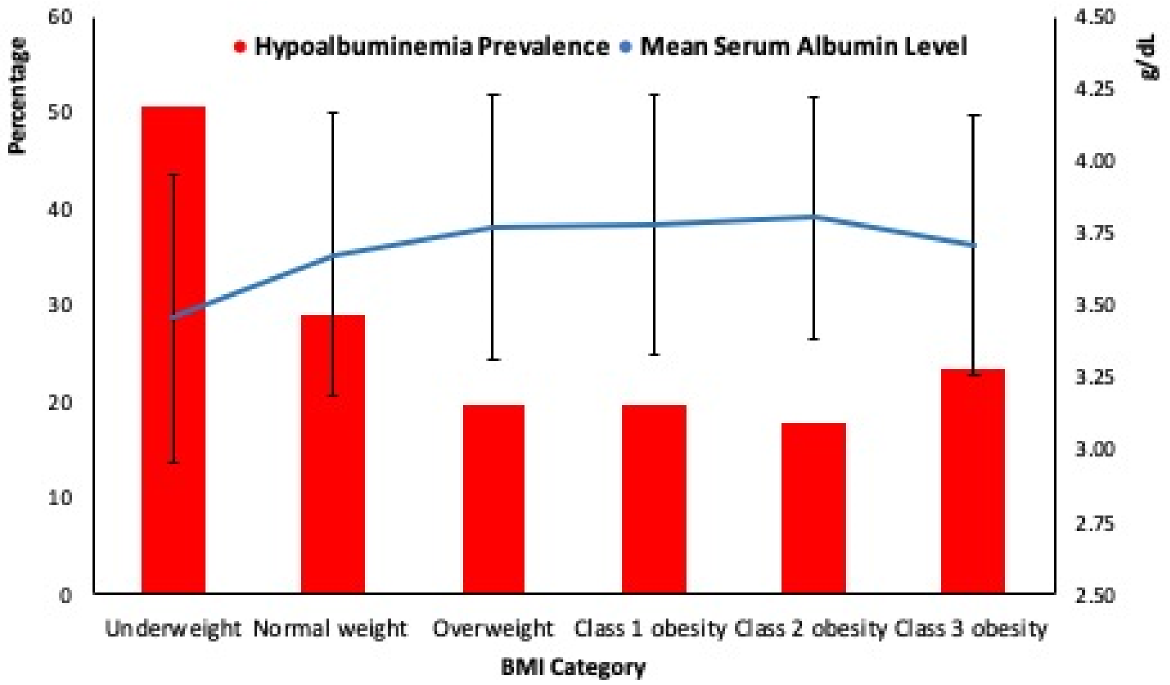

BMI = body mass index.

Supplemental Figure S3. Cumulative Survival

A According to Serum Albumin Status

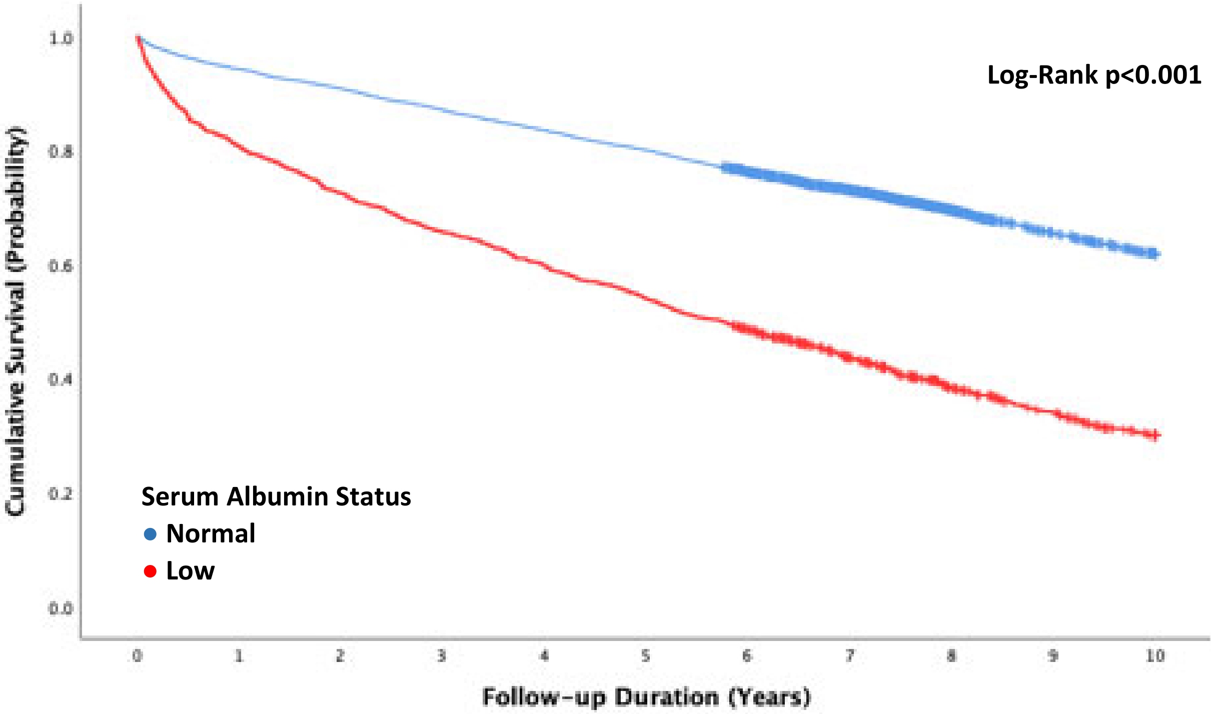

No. at Risk

●	4,858	4,578	4,410	4,229	4,056	3,885	3,640	3,181	2,677	2,361	2,231
●	1,425	1,150	1,032	936	852	769	679	541	420	348	308

B According to Body Mass Index Category

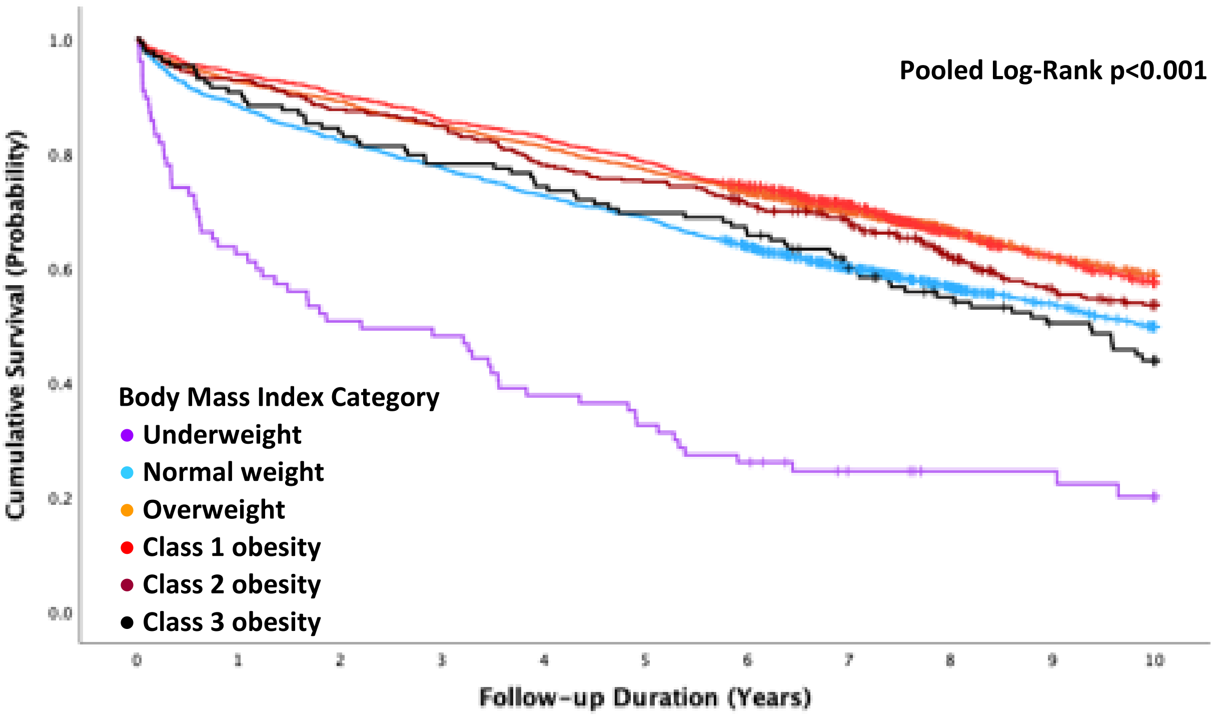

No. at Risk

●	77	48	39	37	29	25	20	14	11	11	9
●	1,825	1,612	1,499	1,414	1,324	1,253	1,143	966	798	699	648
●	2,578	2,384	2,296	2,184	2,093	1,986	1,856	1,605	1,344	1,176	1,117
●	1,303	1,223	1,175	1,115	1,077	1,022	957	830	688	609	566
●	372	345	326	315	290	279	259	235	194	160	152
●	128	116	107	100	95	89	84	72	62	54	47

Supplemental Figure S4. Serum Albumin Decrease-Associated Risk of All-Cause Mortality at 10 Years After Acute Myocardial Infarction Per Multivariable Analysis in the Total Cohort

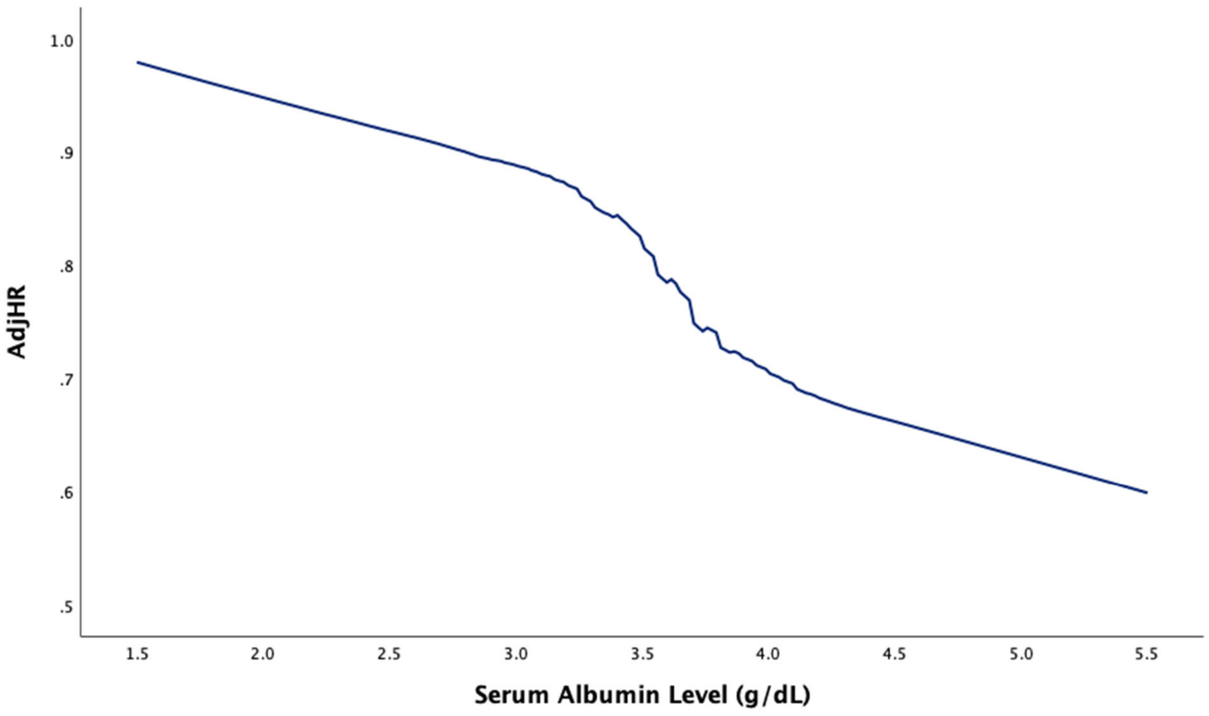

AdjHR = adjusted hazard ratio.

Supplemental Figure S5. Serum Albumin Decrease-Associated Risk of All-Cause Mortality at 10 Years After Acute Myocardial Infarction Per Multivariable Analysis in Each of the Body Mass Index Categories Subgroups

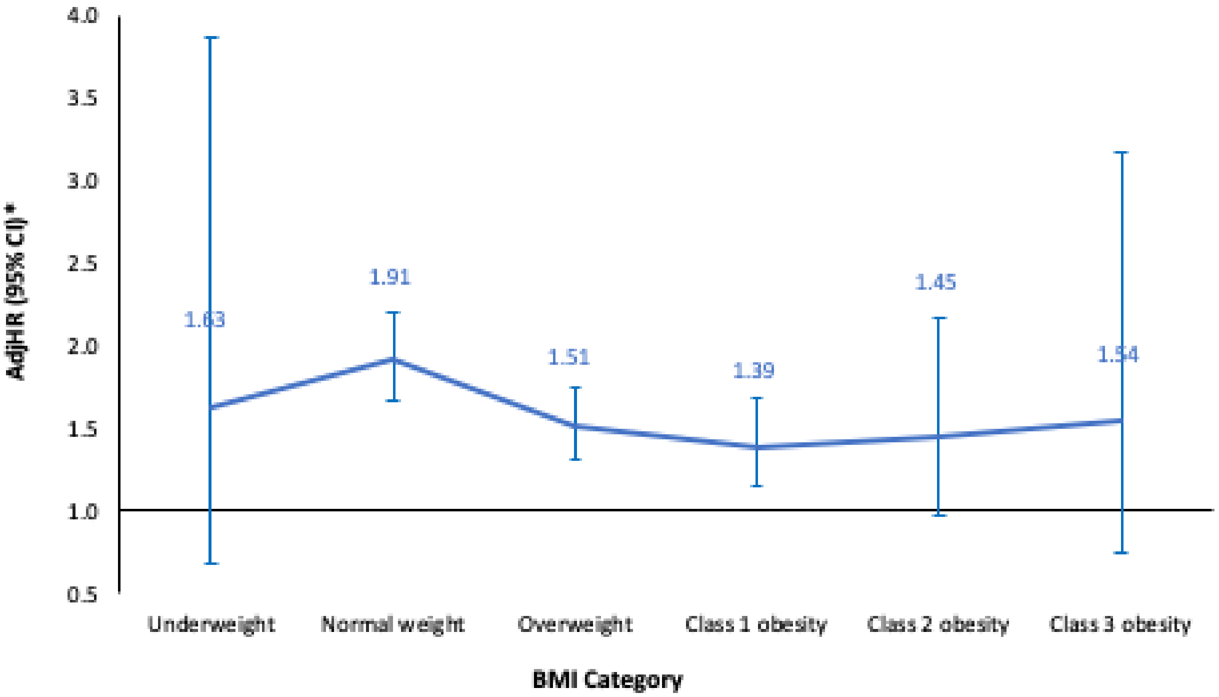

*Per 1 g/dL decrease in serum albumin level. AdjHR = adjusted hazard ratio; BMI = body mass index; CI = confidence interval.

Supplement

Supplemental Table S1. Baseline Clinical Characteristics According to Body Mass Index Category

	Body Mass Index Category						P-Value*
	**Underweight (n=77)**	**Normal Weight (n=1,825)**	**Overweight (n=2,578)**	**Class 1 Obesity (n=1,303)**	**Class 2 Obesity (n=372)**	**Class 3 Obesity (n=128)**	
**Demographic Details**							
Age (years)	69.7± 15.7	65.6±13.7	63.7±12.9	63.2±12.2	62.4±12.5	61.4±13.0	**<0.001†**
Sex Male	51 (66.2)	1,357 (74.4)	1,999 (77.5)	935 (71.8)	243 (65.3)	72 (56.3)	**<0.001**
Non-Jewish Minority	24 (31.2)	366 (20.1)	453 (17.6)	198 (15.2)	54 (14.5)	28 (21.9)	**<0.001**
**Cardiovascular Risk Factors**							
Diabetes Mellitus	24 (31.2)	715 (39.2)	1,089 (42.2)	653 (50.1)	216 (58.1)	85 (66.4)	**<0.001**
Dyslipidemia	46 (59.7)	1,449 (79.4)	2,224 (86.3)	1,145 (87.9)	330 (88.7)	110 (85.9)	**<0.001**
Hypertension	32 (41.6)	858 (47.0)	1,398 (54.2)	819 (62.9)	277 (74.5)	93 (72.7)	**<0.001**
Smoking History	39 (50.6)	897 (49.2)	1,247 (48.4)	605 (46.4)	166 (44.6)	45 (35.2)	**0.027**
Family History of IHD	7 (9.1)	194 (10.6)	331 (12.8)	151 (11.6)	37 (9.9)	5 (3.9)	**0.013**
**Cardiovascular Morbidity**							
Ischemic Heart Disease	53 (68.8)	1,546 (84.7)	2,279 (88.4)	1,165 (89.4)	331 (89)	102 (79.7)	**<0.001**
History of MI	11 (14.3)	298 (16.3)	387 (15.0)	221 (17.0)	55 (14.8)	17 (13.3)	0.559
Prior Revascularization							
PCI	9 (11.7)	299 (16.4)	457 (17.7)	247 (19.0)	69 (18.5)	21 (16.4)	0.331
CABG	8 (10.4)	172 (9.4)	235 (9.1)	129 (9.9)	34 (9.1)	14 (10.9)	0.954
Peripheral Arterial Disease	8 (10.4)	231 (12.7)	301 (11.7)	172 (13.2)	41 (11.0)	14 (10.9)	0.678
Atrial Fibrillation/Flutter	17 (22.1)	277 (15.2)	352 (13.7)	213 (16.3)	72 (19.4)	27 (21.1)	**0.004**
Atrioventricular Block	0 (0.0)	92 (5.0)	90 (3.5)	56 (4.3)	18 (4.8)	3 (2.3)	**0.044**
Clinical Heart Failure	19 (24.7)	334 (18.3)	392 (15.2)	218 (16.7)	61 (16.4)	16 (12.5)	**0.027**
**Non-Cardiovascular Morbidity**							
COPD	14 (18.2)	167 (9.2)	180 (7.0)	117 (9.0)	40 (10.8)	15 (11.7)	**<0.001**
Stage ≥III CKD	14 (18.2)	163 (8.9)	223 (8.7)	120 (9.2)	46 (12.4)	11 (8.6)	**0.023**
Anemia	51 (66.2)	948 (51.9)	1,234 (47.9)	588 (45.1)	160 (43.0)	62 (48.4)	**<0.001**
Neurological Disorders	18 (23.4)	301 (16.5)	368 (14.3)	182 (14.0)	62 (16.7)	18 (14.1)	0.065
Malignancy	13 (16.9)	187 (10.2)	234 (9.1)	125 (9.6)	31 (8.6)	12 (9.4)	0.163
Psychotic Disorders	3 (3.9)	27 (1.5)	35 (1.4)	16 (1.2)	4 (1.1)	1 (0.8)	0.471
Alcohol/Drug Abuse	4 (5.2)	57 (3.1)	39 (1.5)	24 (1.8)	15 (4.0)	1 (0.8)	**<0.001**
**Serum Albumin**							

Mean Level (g/dL)	3.5±0.5	3.7±0.5	3.8±0.5	3.8±0.5	3.8±0.4	3.7±0.4	**<0.001†**
Status							**<0.001**
Normal	38 (49.4)	1296 (71.0)	2073 (80.4)	1047 (80.4)	306 (82.3)	98 (76.6)	
Low	39 (50.6)	529 (29.0)	505 (19.6)	256 (19.6)	66 (17.7)	30 (23.4)	
**Body Mass Index**							
Mean Value (kg/m^2)	17.3±1.3	22.9±1.6	27.4±1.4	32.1±1.4	37.0±1.4	43.3±3.8	**<0.001†**
Category							NA
Underweight	77 (100.0)	0 (0.0)	0 (0.0)	0 (0.0)	0 (0.0)	0 (0.0)	
Normal Weight	0 (0.0)	1,825 (100.0)	0 (0.0)	0 (0.0)	0 (0.0)	0 (0.0)	
Overweight	0 (0.0)	0 (0.0)	2,578 (100.0)	0 (0.0)	0 (0.0)	0 (0.0)	
Class 1 Obesity	0 (0.0)	0 (0.0)	0 (0.0)	1,303 (100.0)	0 (0.0)	0 (0.0)	
Class 2 Obesity	0 (0.0)	0 (0.0)	0 (0.0)	0 (0.0)	372 (100.0)	0 (0.0)	
Class 3 Obesity	0 (0.0)	0 (0.0)	0 (0.0)	0 (0.0)	0 (0.0)	128 (100.0)	

Data are presented as number (percent) or mean±standard deviation. Figures in bold denote statistical significance. * Chi-square test unless stated otherwise; † Analysis of variance test. CABG = coronary artery bypass grafting; CKD = chronic kidney disease; COPD = chronic obstructive pulmonary disease; IHD = ischemic heart disease; MI = myocardial infarction; NA = not applicable; PCI = percutaneous coronary intervention.

Supplemental Table S2. Multivariable Binary Logistic Regression Model for Admission-Time Hypoalbuminemia in the Total Cohort

	AdjOR (95% CI)	P-Value*
**Demographic Details**		
Age (vs <65 years)		
65-74 years	1.19 (1.01-1.41)	**0.039**
≥75 years	1.33 (1.11-1.60)	**0.002**
Sex Male	0.60 (0.52-0.69)	**<0.001**
Non-Jewish Minority	1.54 (1.30-1.83)	**<0.001**
**Cardiovascular Risk Factors**		
Dyslipidemia	0.69 (0.58-0.81)	**<0.001**
**Cardiovascular Morbidity**		
Prior Revascularization		
Percutaneous Coronary Intervention	0.83 (0.69-0.99)	**0.037**
Coronary Artery Bypass Grafting	0.74 (0.58-0.93)	**0.010**
Peripheral Arterial Disease	1.39 (1.15-1.68)	**<0.001**
Atrial Fibrillation/Flutter	1.24 (1.04-1.47)	**0.016**
Clinical Heart Failure	1.32 (1.12-1.56)	**0.001**
**Non-Cardiovascular Morbidity**		
Chronic Obstructive Pulmonary Disease	1.31 (1.06-1.63)	**0.013**
Stage ≥III Chronic Kidney Disease	1.54 (1.26-1.89)	**<0.001**
Anemia	2.05 (1.77-2.37)	**<0.001**
Neurological Disorders	1.38 (1.17-1.64)	**<0.001**
Malignancy	1.37 (1.02-1.85)	**0.037**
**Clinical Presentation**		
Non-ST Elevation vs ST-Elevation Myocardial Infarction	0.77 (0.67-0.88)	**<0.001**

Figures in bold denote statistical significance. * Binary logistic regression analysis. AdjOR = adjusted odds ratio; CI = confidence interval; NA = not applicable.

Supplemental Table S3. Baseline Clinical Characteristics of Patients with Underweight, Normal Weight, and Overweight, Stratified by Serum Albumin Status

	**Underweight**			**Normal Weight**			**Overweight**		
	**Serum Albumin Status**		**P-Value***	**Serum Albumin Status**		**P-Value***	**Serum Albumin Status**		**P-Value***
	**Normal (n=38)**	**Low (n=39)**		**Normal (n=1,296)**	**Low (n=529)**		**Normal (n=2,073)**	**Low (n=505)**	
**Demographic Details**									
Age (years)	66.1±16.6	73.1±14.1	0.050†	63.6±13.7	70.6±12.4	**<0.001†**	62.9±12.9	65.0±12.3	**<0.001†**
Sex Male	26 (68.4)	25 (64.1)	0.689	1,011 (78.0)	346 (65.4)	**<0.001**	1,649 (79.5)	350 (69.3)	**<0.001**
Non-Jewish Minority	9 (23.7)	15 (38.5)	0.162	247 (19.1)	119 (22.5)	0.096	365 (17.6)	88 (17.4)	0.923
**Cardiovascular Risk Factors**									
Diabetes Mellitus	9 (23.7)	15 (38.5)	0.162	463 (35.7)	252 (47.6)	**<0.001**	822 (39.7)	267 (52.9)	**<0.001**
Dyslipidemia	25 (65.8)	21 (53.8)	0.285	1,063 (82.0)	386 (73.0)	**<0.001**	1,819 (87.7)	405 (80.2)	**<0.001**
Hypertension	21 (55.3)	11 (28.2)	**0.016**	581 (44.8)	277 (52.4)	**0.003**	1,127 (54.4)	271 (53.7)	0.776
Smoking History	22 (57.9)	17 (43.6)	0.209	662 (51.1)	235 (44.4)	**0.010**	1,062 (51.2)	185 (36.6)	**<0.001**
Family History of IHD	4 (10.5)	3 (7.7)	0.665	161 (12.4)	33 (6.2)	**<0.001**	297 (14.3)	34 (6.7)	**<0.001**
**Cardiovascular Morbidity**									
Ischemic Heart Disease	31 (81.6)	22 (56.4)	**0.017**	1,133 (87.4)	413 (78.1)	**<0.001**	1,856 (89.5)	423 (83.8)	**<0.001**
History of MI	7 (18.4)	4 (10.3)	0.306	203 (15.7)	95 (18.0)	0.229	299 (14.4)	88 (17.4)	0.090
Prior Revascularization									
PCI	8 (21.1)	1 (2.6)	**0.012**	215 (16.6)	84 (15.9)	0.710	374 (18.0)	83 (16.4)	0.397
CABG	6 (15.8)	2 (5.1)	0.125	121 (9.3)	51 (9.6)	0.840	185 (8.9)	50 (9.9)	0.494
Peripheral Arterial Disease	4 (10.5)	4 (10.3)	0.969	138 (10.6)	93 (17.6)	**<0.001**	216 (10.4)	85 (16.8)	**<0.001**
Atrial Fibrillation/Flutter	3 (7.9)	14 (35.9)	**0.003**	166 (12.8)	111 (21.0)	**<0.001**	244 (11.8)	108 (21.4)	**<0.001**
Atrioventricular Block	0 (0.0)	0 (0.0)	NA	52 (4.0)	40 (7.6)	**0.002**	67 (3.2)	23 (4.6)	0.147
Clinical Heart Failure	10 (26.3)	9 (23.1)	0.742	190 (14.7)	144 (27.2)	**<0.001**	262 (12.6)	130 (25.7)	**<0.001**
**Non-Cardiovascular Morbidity**									
COPD	5 (13.2)	9 (23.1)	0.259	94 (7.3)	73 (13.8)	**<0.001**	137 (6.6)	43 (8.5)	0.132
Stage ≥III CKD	5 (13.2)	9 (23.1)	0.259	70 (5.4)	93 (17.6)	**<0.001**	131 (6.3)	92 (18.2)	**<0.001**
Anemia	22 (57.9)	29 (74.4)	0.127	573 (44.2)	375 (70.9)	**<0.001**	895 (43.2)	339 (67.1)	**<0.001**
Neurological Disorders	7 (18.4)	11 (28.2)	0.310	171 (13.2)	130 (24.6)	**<0.001**	262 (12.6)	106 (21.0)	**<0.001**
Malignancy	2 (5.3)	3 (7.7)	0.665	46 (3.5)	38 (7.2)	**0.001**	56 (2.7)	33 (6.5)	**<0.001**
Psychotic Disorders	1 (2.6)	2 (5.1)	0.571	17 (1.3)	10 (1.9)	0.353	27 (1.3)	8 (1.6)	0.624

Alcohol/Drug Abuse	3 (7.9)	1 (2.6)	0.292	45 (3.5)	12 (2.3)	0.180	24 (1.2)	15 (3.0)	**0.003**
**Serum Albumin**									
Mean Level (g/dL)	3.9±0.3	3.1±0.3	**<0.001**†	3.9±0.3	3.1±0.3	**<0.001**†	3.9±0.3	3.1±0.3	**<0.001**†
**Body Mass Index**									
Mean Value (kg/m^2)	17.5±1.1	17.1±1.4	0.101†	23.0±1.7	22.5±1.7	**<0.001**†	27.4±1.4	27.3±1.4	**0.047**†

Data are presented as number (percent) or mean±standard deviation. Figures in bold denote statistical significance. * Chi-square test unless stated otherwise; † Student's t-test. CABG = coronary artery bypass grafting; CKD = chronic kidney disease; COPD = chronic obstructive pulmonary disease; IHD = ischemic heart disease; MI = myocardial infarction; NA = not applicable; PCI = percutaneous coronary intervention.

Supplemental Table S4. Baseline Clinical Characteristics of Patients with Obesity, Stratified by Serum Albumin Status

	Class 1 Obesity			Class 2 Obesity			Class 3 Obesity		
	**Serum Albumin Status**		**P-Value***	**Serum Albumin Status**		**P-Value***	**Serum Albumin Status**		**P-Value***
	**Normal (n=1,047)**	**Low (n=256)**		**Normal (n=306)**	**Low (n=66)**		**Normal (n=98)**	**Low (n=30)**	
**Demographic Details**									
Age (years)	62.0±11.9	68.0±12.2	**<0.001†**	61.7±12.2	65.7±13.5	**0.019†**	60.4±13.6	64.5±10.4	0.128†
Sex Male	797 (76.1)	138 (53.9)	**<0.001**	211 (69.0)	32 (48.5)	**0.002**	59 (60.2)	13 (43.3)	0.103
Non-Jewish Minority	162 (15.5)	36 (14.1)	0.573	39 (12.7)	15 (22.7)	**0.037**	20 (20.4)	8 (26.7)	0.468
**Cardiovascular Risk Factors**									
Diabetes Mellitus	513 (49.0)	140 (54.7)	0.103	170 (55.6)	46 (69.7)	**0.035**	63 (64.3)	22 (73.3)	0.359
Dyslipidemia	934 (89.2)	211 (82.4)	**0.003**	277 (90.5)	53 (80.3)	**0.017**	84 (85.7)	26 (86.7)	0.896
Hypertension	659 (62.9)	160 (62.5)	0.896	227 (74.2)	50 (75.8)	0.790	68 (69.4)	25 (83.3)	0.134
Smoking History	518 (49.5)	87 (34.0)	**<0.001**	145 (47.4)	21 (31.8)	**0.021**	37 (37.8)	8 (26.7)	0.266
Family History of IHD	130 (12.4)	21 (8.2)	0.059	32 (10.5)	5 (7.6)	0.478	3 (3.1)	2 (6.7)	0.372
**Cardiovascular Morbidity**									
Ischemic Heart Disease	949 (90.6)	216 (84.4)	**0.004**	280 (91.5)	51 (77.3)	0.001	79 (80.6)	23 (76.7)	0.638
History of MI	171 (16.3)	50 (19.5)	0.222	44 (14.4)	11 (16.7)	0.635	15 (15.3)	2 (6.7)	0.222
Prior Revascularization									
PCI	196 (18.7)	51 (19.9)	0.660	59 (19.3)	10 (15.2)	0.434	17 (17.3)	4 (13.3)	0.603
CABG	103 (9.8)	26 (10.2)	0.878	30 (9.8)	4 (6.1)	0.339	11 (11.2)	3 (10.0)	0.851
Peripheral Arterial Disease	123 (11.7)	49 (19.1)	**0.002**	30 (9.8)	11 (16.7)	0.106	7 (7.1)	7 (23.3)	**0.013**
Atrial Fibrillation/Flutter	159 (15.2)	54 (21.1)	**0.022**	50 (16.3)	22 (33.3)	**0.002**	20 (20.4)	7 (23.3)	0.731
Atrioventricular Block	45 (4.3)	11 (4.3)	0.999	14 (4.6)	4 (6.1)	0.610	3 (3.1)	0 (0.0)	0.332
Clinical Heart Failure	138 (13.2)	80 (31.3)	**<0.001**	45 (14.7)	16 (24.2)	0.058	12 (12.2)	4 (13.3)	0.875
**Non-Cardiovascular Morbidity**									
COPD	81 (7.7)	36 (14.1)	**0.002**	27 (8.8)	13 (19.7)	**0.010**	11 (11.2)	4 (13.3)	0.753
Stage ≥III CKD	79 (7.5)	41 (16.0)	**<0.001**	29 (9.5)	17 (25.8)	**<0.001**	8 (8.2)	3 (10.0)	0.753
Anemia	422 (40.3)	166 (64.8)	**<0.001**	115 (37.6)	45 (68.2)	**<0.001**	39 (39.8)	23 (76.7)	**<0.001**
Neurological Disorders	131 (12.5)	51 (19.9)	**0.002**	45 (14.7)	17 (25.8)	**0.029**	8 (8.2)	10 (33.3)	**0.001**
Malignancy	46 (4.4)	9 (3.5)	0.531	7 (2.3)	2 (3.0)	0.722	2 (2.0)	2 (6.7)	0.234
Psychotic Disorders	8 (0.8)	8 (3.1)	**0.002**	2 (0.7)	2 (3.0)	0.090	1 (1.0)	0 (0.0)	1.000

Alcohol/Drug Abuse	20 (1.9)	4 (1.6)	0.711	15 (4.9)	0 (0.0)	0.066	1 (1.0)	0 (0.0)	1.000
**Serum Albumin**									
Mean Level (g/dL)	3.9±0.3	3.1±0.3	**<0.001**†	4.0±0.3	3.1±0.3	**<0.001**†	3.9±0.3	3.1±0.3	**<0.001**†
**Body Mass Index**									
Mean Value (kg/m^2)	32.0±1.4	32.1±1.4	0.529†	37.0±1.4	37.1±1.6	0.454†	43.4±4.0	43.3±3.0	0.981†

Data are presented as number (percent) or mean±standard deviation. Figures in bold denote statistical significance. * Chi-square test unless stated otherwise; † Student's t-test.

CABG = coronary artery bypass grafting; CKD = chronic kidney disease; COPD = chronic obstructive pulmonary disease; IHD = ischemic heart disease; MI = myocardial infarction; NA = not applicable; PCI = percutaneous coronary intervention.

Supplemental Table S5. Acute Event Aspects According to Body Mass Index Category

	**Body Mass Index Category**						**P-Value***
	**Underweight (n=77)**	**Normal Weight (n=1,825)**	**Overweight (n=2,578)**	**Class 1 Obesity (n=1,303)**	**Class 2 Obesity (n=372)**	**Class 3 Obesity (n=128)**	
**Clinical Presentation**							
Cardiac Arrest	0 (0.0)	8 (0.4)	11 (0.4)	6 (0.5)	2 (0.5)	1 (0.8)	0.692
Cardiogenic Shock	3 (3.9)	40 (2.2)	48 (1.9)	9 (0.7)	6 (1.6)	0 (0.0)	**0.003**
ST-Elevation MI	33 (42.9)	832 (45.6)	1,191 (46.2)	556 (42.7)	131 (35.2)	41 (32.0)	**<0.001**
**Echocardiographic Parameters**							
Echocardiogram Performed	57 (74.0)	1,471 (80.6)	2,156 (83.6)	1,094 (84.0)	307 (82.5)	101 (78.9)	**<0.001**
Severe LV Dysfunction	19 (33.3)	214 (14.5)	255 (11.8)	99 (9.0)	29 (9.4)	4 (4.0)	**0.017**
LV Hypertrophy	0 (0.0)	51 (3.5)	122 (5.7)	82 (7.5)	32 (10.4)	13 (12.9)	**<0.001**
Mitral Regurgitation	7 (12.3)	121 (8.2)	125 (5.8)	55 (5.0)	9 (2.9)	2 (2.0)	**<0.001**
Tricuspid Regurgitation	5 (8.8)	69 (4.7)	71 (3.3)	37 (3.4)	4 (1.3)	3 (3.0)	**0.011**
Pulmonary Hypertension	8 (14.0)	113 (7.7)	161 (7.5)	85 (7.8)	20 (6.5)	11 (10.9)	0.363
**Angiographic Parameters**							
Angiogram Performed	45 (58.4)	1,344 (73.6)	1,996 (77.4)	1,027 (78.8)	286 (76.9)	95 (74.2)	**<0.001**
Vessels Significantly Involved							0.285
0	4 (8.9)	52 (3.9)	65 (3.3)	29 (2.8)	11 (3.8)	7 (7.4)	
1	12 (26.7)	320 (23.8)	451 (22.6)	229 (22.3)	71 (24.8)	23 (24.2)	
2	12 (26.7)	341 (25.4)	552 (27.7)	283 (27.6)	67 (23.4)	29 (30.5)	
3 / Left Main	17 (37.8)	631 (46.9)	928 (46.5)	486 (47.3)	137 (47.9)	36 (37.9)	
**Hospital Course**							
Revascularization Approach							**<0.001**
No / Conservative Treatment	29 (37.7)	354 (19.4)	393 (15.2)	198 (15.2)	64 (17.2)	25 (19.5)	
PCI	41 (53.2)	1,075 (58.9)	1,546 (60.0)	770 (59.1)	221 (59.4)	84 (65.6)	
CABG	7 (9.1)	396 (21.7)	639 (24.8)	335 (25.7)	87 (23.4)	19 (14.8)	
Intra-Aortic Balloon Pulsation	2 (2.6)	46 (2.5)	87 (3.4)	29 (2.2)	7 (1.9)	1 (0.8)	0.465
Any Form of Pacing	1 (1.3)	42 (2.3)	55 (2.1)	26 (2.0)	10 (2.7)	2 (1.6)	0.746
Mechanical Ventilation	6 (7.8)	79 (4.3)	92 (3.6)	57 (4.4)	24 (6.5)	6 (4.7)	0.524
Gastrointestinal Bleeding	2 (2.6)	44 (2.4)	60 (2.3)	32 (2.5)	8 (2.2)	1 (0.8)	0.906
Blood Transfusion	18 (23.4)	325 (17.8)	417 (16.2)	192 (14.7)	49 (13.2)	18 (14.1)	**0.003**
Sepsis	1 (1.3)	26 (1.4)	32 (1.2)	18 (1.4)	4 (1.1)	3 (2.3)	0.956
Intensive Care Unit Stay	47 (61.0)	1,331 (72.9)	1,951 (75.7)	986 (75.7)	278 (74.7)	82 (64.1)	**0.001**

Hospitalization Length (days)	11.4±11.3	11.2±9.4	11.3±10.1	11.5±9.9	11.7±9.8	11.0±9.7	0.936†

Data are presented as number (percent) or mean±standard deviation, as appropriate. Figures in bold denote statistical significance. * Chi-square test unless stated otherwise; † Analysis of variance test. CABG = coronary artery bypass grafting; LV = left ventricular; MI = myocardial infarction; PCI = percutaneous coronary intervention.

Supplemental Table S6. Acute Event Aspects in Patients with Underweight, Normal Weight, and Overweight, Stratified by Serum Albumin Status

	**Underweight**			**Normal Weight**			**Overweight**		
	**Serum Albumin Status**		**P-Value***	**Serum Albumin Status**		**P-Value***	**Serum Albumin Status**		**P-Value***
	**Normal (n=38)**	**Low (n=39)**		**Normal (n=1,296)**	**Low (n=529)**		**Normal (n=2,073)**	**Low (n=505)**	
**Clinical Presentation**									
Cardiac Arrest	0 (0.0)	0 (0.0)	NA	3 (0.2)	5 (0.9)	0.050	7 (0.3)	4 (0.8)	0.152
Cardiogenic Shock	0 (0.0)	3 (7.7)	0.240	13 (1.0)	27 (5.1)	**<0.001**	28 (1.4)	20 (4.0)	**<0.001**
ST-Elevation MI	18 (47.4)	15 (38.5)	0.430	613 (47.3)	219 (41.4)	**0.022**	968 (46.7)	223 (44.2)	0.305
**Echocardiographic Parameters**									
Echocardiogram Performed	29 (76.3)	28 (71.8)	0.651	1082 (83.5)	389 (73.5)	**<0.001**	1746 (84.2)	410 (81.2)	0.098
Severe LV Dysfunction	7 (24.1)	12 (42.9)	0.134	117 (10.8)	97 (24.9)	**<0.001**	176 (10.1)	79 (19.3)	**<0.001**
LV Hypertrophy	0 (0.0)	0 (0.0)	NA	35 (3.2)	16 (4.1)	0.417	86 (4.9)	36 (8.8)	**0.002**
Mitral Regurgitation	4 (13.8)	3 (10.7)	1.000	61 (5.6)	60 (15.4)	**<0.001**	84 (4.8)	41 (10.0)	**<0.001**
Tricuspid Regurgitation	3 (10.3)	2 (7.1)	1.000	36 (3.3)	33 (8.5)	**<0.001**	44 (2.5)	27 (6.6)	**<0.001**
Pulmonary Hypertension	6 (20.7)	2 (7.1)	0.253	66 (6.1)	47 (12.1)	**<0.001**	100 (5.7)	61 (14.9)	**<0.001**
**Angiographic Parameters**									
Angiogram Performed	28 (73.7)	17 (43.6)	**0.007**	1017 (78.5)	327 (61.8)	**<0.001**	1650 (79.6)	346 (68.5)	**<0.001**
Vessels Significantly Involved			0.822			0.470			**0.002**
0	2 (7.1)	2 (11.8)		41 (4.0)	11 (3.4)		51 (3.1)	14 (4.0)	
1	7 (25.0)	5 (29.4)		250 (24.6)	70 (21.4)		390 (23.6)	61 (17.6)	
2	7 (25.0)	5 (29.4)		260 (25.6)	81 (24.8)		472 (28.6)	80 (23.1)	
3 / Left Main	12 (42.9)	5 (29.4)		466 (45.8)	165 (50.5)		737 (44.7)	191 (55.2)	
**Hospital Course**									
Revascularization Approach			**0.001**			**<0.001**			**<0.001**
No / Conservative Treatment	7 (18.4)	22 (56.4)		190 (14.7)	164 (31.0)		270 (13.0)	123 (24.4)	
PCI	25 (65.8)	16 (41.0)		814 (62.8)	261 (49.3)		1296 (62.5)	250 (49.5)	
CABG	6 (15.8)	1 (2.6)		292 (22.5)	104 (19.7)		507 (24.5)	132 (26.1)	
Intra-Aortic Balloon Pulsation	0 (0.0)	2 (5.1)	0.494	16 (1.2)	30 (5.7)	**<0.001**	47 (2.3)	40 (7.9)	**<0.001**
Any Form of Pacing	0 (0.0)	1 (2.6)	1.000	18 (1.4)	24 (4.5)	**<0.001**	39 (1.9)	16 (3.2)	0.073
Mechanical Ventilation	3 (7.9)	3 (7.7)	1.000	27 (2.1)	52 (9.8)	**<0.001**	43 (2.1)	49 (9.7)	**<0.001**
Gastrointestinal Bleeding	0 (0.0)	2 (5.1)	0.157	26 (2.0)	18 (3.4)	0.078	30 (1.4)	30 (5.9)	**<0.001**

Blood Transfusion	7 (18.4)	11 (28.2)	0.310	189 (14.6)	136 (25.7)	**<0.001**	279 (13.5)	138 (27.3)	**<0.001**
Sepsis	0 (0.0)	1 (2.6)	1.000	6 (0.5)	20 (3.8)	**<0.001**	10 (0.5)	22 (4.4)	**<0.001**
Intensive Care Unit Stay	29 (76.3)	18 (46.2)	**0.007**	987 (76.2)	344 (65.0)	**<0.001**	1592 (76.8)	359 (71.1)	**0.007**
Hospitalization Length (days)	9.4±5.3	13.4±14.8	0.112†	10.2±8.0	13.7±11.8	**<0.001**†	10.4±8.5	15.2±14.5	**<0.001**†

Data are presented as number (percent) or mean±standard deviation, as appropriate. Figures in bold denote statistical significance. * Chi-square test unless stated otherwise; † Student's t-test. CABG = coronary artery bypass grafting; LV = left ventricular; MI = myocardial infarction; NA = not applicable; PCI = percutaneous coronary intervention.

Supplemental Table S7. Acute Event Aspects in Patients with Obesity, Stratified by Serum Albumin Status

	Class 1 Obesity			Class 2 Obesity			Class 3 Obesity		
	Serum Albumin Status		P-Value*	Serum Albumin Status		P-Value*	Serum Albumin Status		P-Value*
	Normal (n=1,047)	Low (n=256)		Normal (n=306)	Low (n=66)		Normal (n=98)	Low (n=30)	
**Clinical Presentation**									
Cardiac Arrest	4 (0.4)	2 (0.8)	0.336	0 (0.0)	2 (3.0)	**0.031**	1 (1.0)	0 (0.0)	0.766
Cardiogenic Shock	6 (0.6)	3 (1.2)	0.391	1 (0.3)	5 (7.6)	**<0.001**	0 (0.0)	0 (0.0)	NA
ST-Elevation MI	452 (43.2)	104 (40.6)	0.460	106 (34.6)	25 (37.9)	0.617	30 (30.6)	11 (36.7)	0.534
**Echocardiographic Parameters**									
Echocardiogram Performed	894 (85.4)	200 (78.1)	**0.005**	260 (85.0)	47 (71.2)	**0.008**	83 (84.7)	18 (60.0)	**0.004**
Severe LV Dysfunction	70 (7.8)	29 (14.5)	**0.003**	22 (8.5)	7 (14.9)	0.176	4 (4.8)	0 (0.0)	1.000
LV Hypertrophy	65 (7.3)	17 (8.5)	0.551	28 (10.8)	4 (8.5)	0.798	10 (12.0)	3 (16.7)	0.697
Mitral Regurgitation	37 (4.1)	18 (9.0)	**0.004**	8 (3.1)	1 (2.1)	1.000	2 (2.4)	0 (0.0)	1.000
Tricuspid Regurgitation	25 (2.8)	12 (6.0)	**0.023**	3 (1.2)	1 (2.1)	0.487	2 (2.4)	1 (5.6)	0.449
Pulmonary Hypertension	54 (6.0)	31 (15.5)	**<0.001**	17 (6.5)	3 (6.4)	1.000	9 (10.8)	2 (11.1)	1.000
**Angiographic Parameters**									
Angiogram Performed	863 (82.4)	164 (64.1)	**<0.001**	243 (79.4)	43 (65.2)	**0.013**	79 (80.6)	16 (53.3)	**0.003**
Vessels Significantly Involved			0.157			0.174			0.659
0	23 (2.7)	6 (3.7)		8 (3.3)	3 (7.0)		7 (8.9)	0 (0.0)	
1	203 (23.5)	26 (15.9)		60 (24.7)	11 (25.6)		19 (24.1)	4 (25.0)	
2	237 (27.5)	46 (28.0)		62 (25.5)	5 (11.6)		24 (30.4)	5 (31.3)	
3 / Left Main	400 (46.3)	86 (52.4)		113 (46.5)	24 (55.8)		29 (36.7)	7 (43.8)	
**Hospital Course**									
Revascularization Approach			**<0.001**			**0.002**			**0.025**
No / Conservative Treatment	121 (11.6)	77 (30.1)		44 (14.4)	20 (30.3)		14 (14.3)	11 (36.7)	
PCI	650 (62.1)	120 (46.9)		193 (63.1)	28 (42.4)		69 (70.4)	15 (50.0)	
CABG	276 (26.4)	59 (23.0)		69 (22.5)	18 (27.3)		15 (15.3)	4 (13.3)	
Intra-Aortic Balloon Pulsation	16 (1.5)	13 (5.1)	**<0.001**	3 (1.0)	4 (6.1)	**0.021**	0 (0.0)	1 (3.3)	0.234
Any Form of Pacing	20 (1.9)	6 (2.3)	0.657	6 (2.0)	4 (6.1)	0.082	0 (0.0)	2 (6.7)	0.054
Mechanical Ventilation	36 (3.4)	21 (8.2)	**<0.001**	11 (3.6)	13 (19.7)	**<0.001**	2 (2.0)	4 (13.3)	**0.027**
Gastrointestinal Bleeding	16 (1.5)	16 (6.3)	**<0.001**	4 (1.3)	4 (6.1)	**0.016**	0 (0.0)	1 (3.3)	0.234
Blood Transfusion	130 (12.4)	62 (24.2)	**<0.001**	36 (11.8)	13 (19.7)	0.084	8 (8.2)	10 (33.3)	**0.001**

Sepsis	6 (0.6)	12 (4.7)	**<0.001**	0 (0.0)	4 (6.1)	<0.001	1 (1.0)	2 (6.7)	0.137
Intensive Care Unit Stay	823 (78.6)	163 (63.7)	**<0.001**	230 (75.2)	48 (72.7)	0.680	66 (67.3)	16 (53.3)	0.162
Hospitalization Length (days)	10.9±9.0	13.9±12.5	**<0.001**†	11.1±9.6	14.3±10.2	**0.014**†	9.9±8.7	14.5±12.0	0.058†

Data are presented as number (percent) or mean±standard deviation, as appropriate. Figures in bold denote statistical significance. * Chi-square test unless stated otherwise; † Student's t-test. CABG = coronary artery bypass grafting; LV = left ventricular; MI = myocardial infarction; NA = not applicable; PCI = percutaneous coronary intervention.

Supplemental Table S8. Ten-Year All-Cause Mortality

		Incidence				Risk Associated with Low Serum Albumin Status*	
		Total Cohort	Serum Albumin Status		P-Value†	HR (95% CI)	P-Value¶
			Normal	Low			
**Total Cohort**		2,669/6,283 (42.5)	1,725/4,858 (35.5)	944/1,425 (66.2)	**<0.001**	2.62 (2.42-2.84)	**<0.001**
**Body Mass Index Category**	**Underweight**	60/77 (77.9)	25/38 (65.8)	35/39 (89.7)	**0.011**	1.98 (1.18-3.33)	**0.010**
	**Normal Weight**	867/1,825 (47.5)	487/1,296 (37.6)	380/529 (71.8)	**<0.001**	2.80 (2.45-3.21)	**<0.001**
	**Overweight**	994/2,578 (38.6)	680/2,073 (32.8)	314/505 (62.2)	**<0.001**	2.55 (2.23-2.92	**<0.001**
	**Class 1 Obesity**	516/1,303 (39.6)	364/1,047 (34.8)	152/256 (59.4)	**<0.001**	2.33 (1.92-2.81)	**<0.001**
	**Class 2 Obesity**	163/372 (43.8)	123/306 (40.2)	40/66 (60.6)	**0.002**	1.88 (1.31-2.69)	**<0.001**
	**Class 3 Obesity**	69/128 (53.9)	44/98 (46.9)	23/30 (76.7)	**0.004**	2.16 (1.30-3.57)	**0.003**
**P-Value†**		**<0.001**	0.808	**<0.001**			

Data are presented as number (percent). Figures in bold denote statistical significance.

* According to univariable analysis; †Student's t-test; ¶ Cox regression analysis. CI = confidence interval; HR = hazard ratio; NA = not applicable.

Supplemental Table S9. Multivariable Cox Proportional Hazard Models for the Outcome of All-Cause Mortality at 10 Years in Patients with Underweight, Normal Weight, and Overweight

	Underweight		Normal Weight		Overweight	
	**AdjHR (95% CI)**	**P-Value***	**AdjHR (95% CI)**	**P-Value***	**AdjHR (95% CI)**	**P-Value***
**Demographic Details**						
Age (vs <65 years)						
65-74 years	2.17 (0.76-6.19)	0.148	2.18 (1.79-2.65)	**<0.001**	1.74 (1.46-2.07)	**<0.001**
≥75 years	4.29 (1.83-10.04)	**<0.001**	3.61 (2.98-4.37)	**<0.001**	2.89 (2.44-3.43)	**<0.001**
**Cardiovascular Risk Factors**						
Diabetes Mellitus	2.15 (0.87-5.28)	0.096	1.38 (1.2-1.59)	**<0.001**	1.49 (1.30-1.70)	**<0.001**
Dyslipidemia	0.61 (0.29-1.32)	0.210	0.85 (0.72-1.00)	0.051	0.94 (0.8-1.12)	0.514
Family History of Ischemic Heart Disease	1.22 (0.33-4.55)	0.764	0.67 (0.45-1.00)	0.051	0.51 (0.36-0.74)	**<0.001**
**Cardiovascular Morbidity**						
History of Myocardial Infarction	0.94 (0.28-3.19)	0.917	1.32 (1.12-1.56)	**<0.001**	1.11 (0.94-1.30)	0.211
Peripheral Arterial Disease	2.11 (0.72-6.19)	0.173	1.29 (1.07-1.54)	**0.007**	1.34 (1.13-1.58)	**<0.001**
Atrial Fibrillation/Flutter	0.72 (0.28-1.88)	0.505	1.26 (1.07-1.49)	**0.007**	1.50 (1.29-1.75)	**<0.001**
Clinical Heart Failure	0.39 (0.17-0.94)	**0.036**	1.20 (1.02-1.42)	**0.030**	1.17 (1.00-1.37)	0.056
**Non-Cardiovascular Morbidity**						
Chronic Obstructive Pulmonary Disease	2.80 (0.92-8.58)	0.071	1.78 (1.46-2.17)	**<0.001**	1.67 (1.37-2.03)	**<0.001**
Stage ≥III Chronic Kidney Disease	1.97 (0.84-4.63)	0.120	1.73 (1.42-2.11)	**<0.001**	1.83 (1.54-2.18)	**<0.001**
Anemia	1.98 (0.83-4.72)	0.123	1.52 (1.30-1.77)	**<0.001**	1.37 (1.19-1.58)	**<0.001**
Neurological Disorders	1.95 (0.92-4.12)	0.082	1.53 (1.31-1.80)	**<0.001**	1.54 (1.32-1.80)	**<0.001**
Malignancy	9.87 (2.80-34.86)	**<0.001**	1.66 (1.28-2.14)	**<0.001**	1.76 (1.35-2.28)	**<0.001**
Alcohol/Drug abuse	1.34 (0.31-5.76)	0.695	1.75 (1.19-2.57)	**0.005**	1.57 (0.95-2.59)	0.080
**Clinical Presentation**						
Non-ST Elevation vs ST-Elevation Myocardial Infarction	1.07 (0.47-2.50)	0.855	0.76 (1.15-1.59)	**<0.001**	0.9 (0.93-1.23)	0.343
**Echocardiographic Parameters**						
Severe Left Ventricular Dysfunction	1.81 (0.74-4.41)	0.191	1.25 (1.02-1.53)	**0.031**	1.56 (1.28-1.88)	**<0.001**
Left Ventricular Hypertrophy	NA	NA	1.33 (0.92-1.91)	0.126	1.39 (1.07-1.79)	**0.013**
Tricuspid Regurgitation	3.45 (0.84-14.15)	0.085	1.22 (0.90-1.67)	0.201	1.29 (0.94-1.75)	0.115
Pulmonary Hypertension	1.61 (0.37-7.01)	0.525	0.98 (0.76-1.28)	0.894	1.18 (0.91-1.53)	0.217
**Hospital Course**						

Revascularization Approach (vs Conservative):						
Percutaneous Coronary Intervention	0.27 (0.11-0.67)	**0.005**	0.57 (0.48-0.68)	**<0.001**	0.53 (0.45-0.62)	**<0.001**
Coronary Artery Bypass Grafting	0.44 (0.12-1.68)	0.231	0.39 (0.31-0.49)	**<0.001**	0.35 (0.29-0.43)	**<0.001**
**Serum Albumin Status**						
Low vs Normal Serum Albumin Level	0.84 (0.35-2.00)	0.698	1.73 (1.50-1.99)	**<0.001**	1.55 (1.35-1.79)	**<0.001**

Figures in bold denote statistical significance. * Cox regression analysis. AdjHR = adjusted hazard ratio; CI = confidence interval; NA = not applicable.

Supplemental Table S10. Multivariable Cox Proportional Hazard Models for the Outcome of All-Cause Mortality at 10 Years in Patients with Obesity

	Class 1 Obesity		Class 2 Obesity		Class 3 Obesity	
	AdjHR (95% CI)	P-Value*	AdjHR (95% CI)	P-Value*	AdjHR (95% CI)	P-Value*
**Demographic Details**						
Age (vs <65 years)						
65-74 years	2.20 (1.74-2.77)	**<0.001**	2.42 (1.56-3.78)	**<0.001**	4.26 (1.81-10.04)	**<0.001**
≥75 years	2.68 (2.08-3.46)	**<0.001**	5.11 (3.18-8.21)	**<0.001**	5.48 (2.58-11.64)	**<0.001**
**Cardiovascular Risk Factors**						
Diabetes Mellitus	1.24 (1.03-1.50)	**0.027**	1.19 (0.82-1.74)	0.365	2.77 (1.33-5.79)	**0.007**
Dyslipidemia	1.09 (0.84-1.40)	0.534	0.91 (0.55-1.51)	0.713	1.20 (0.52-2.77)	0.663
Family History of Ischemic Heart Disease	0.76 (0.49-1.19)	0.233	1.02 (0.45-2.29)	0.965	1.00 (0.12-8.60)	0.998
**Cardiovascular Morbidity**						
History of Myocardial Infarction	1.22 (0.98-1.51)	0.072	1.42 (0.90-2.24)	0.132	0.68 (0.29-1.61)	0.384
Peripheral Arterial Disease	1.19 (0.95-1.50)	0.137	1.41 (0.90-2.23)	0.138	2.10 (0.96-4.61)	0.064
Atrial Fibrillation/Flutter	1.38 (1.11-1.70)	**0.003**	1.77 (1.22-2.57)	**0.003**	0.74 (0.35-1.56)	0.427
Clinical Heart Failure	1.53 (1.23-1.91)	**<0.001**	1.00 (0.65-1.54)	0.994	1.43 (0.64-3.19)	0.379
**Non-Cardiovascular Morbidity**						
Chronic Obstructive Pulmonary Disease	1.54 (1.19-1.97)	**<0.001**	1.26 (0.77-2.06)	0.365	2.26 (1.04-4.94)	**0.040**
Stage ≥III Chronic Kidney Disease	1.60 (1.25-2.03)	**<0.001**	1.57 (1.01-2.44)	**0.043**	3.06 (1.26-7.44)	**0.013**
Anemia	1.35 (1.10-1.65)	**0.004**	1.43 (0.98-2.08)	0.061	1.51 (0.80-2.85)	0.203
Neurological Disorders	1.59 (1.27-1.99)	**<0.001**	1.57 (1.04-2.36)	**0.031**	2.01 (0.91-4.45)	0.084
Malignancy	1.78 (1.28-2.49)	**<0.001**	5.49 (2.5-12.06)	**<0.001**	1.61 (0.36-7.16)	0.529
Alcohol/Drug abuse	2.08 (1.17-3.68)	**0.013**	2.46 (1.07-5.68)	**0.034**	-	-
**Clinical Presentation**						
Non-ST Elevation vs ST-Elevation Myocardial Infarction	0.82 (1.02-1.56)	**0.032**	0.70 (1.04-2.33)	**0.032**	1.46 (1.01-3.70)	**0.048**
**Echocardiographic Parameters**						
Severe Left Ventricular Dysfunction	1.34 (0.99-1.82)	0.055	2.06 (1.16-3.67)	**0.014**	3.25 (0.93-11.37)	0.066
Left Ventricular Hypertrophy	1.35 (0.99-1.86)	0.062	1.06 (0.58-1.96)	0.851	2.93 (1.15-7.46)	**0.024**
Tricuspid Regurgitation	1.21 (0.78-1.88)	0.400	0.77 (0.20-2.96)	0.698	7.75 (1.51-39.85)	**0.014**
Pulmonary Hypertension	1.29 (0.93-1.78)	0.134	0.80 (0.34-1.86)	0.600	1.12 (0.43-2.90)	0.816
**Hospital Course**						

Revascularization Approach (vs Conservative):						
Percutaneous Coronary Intervention	0.60 (0.47-0.76)	**<0.001**	0.78 (0.50-1.20)	0.251	0.73 (0.33-1.59)	0.424
Coronary Artery Bypass Grafting	0.50 (0.38-0.66)	**<0.001**	0.61 (0.37-1.02)	0.061	1.03 (0.42-2.53)	0.956
**Serum Albumin Status**						
Low vs Normal Serum Albumin Level	1.37 (1.12-1.68)	**0.002**	1.17 (0.77-1.75)	0.465	1.70 (0.84-3.42)	0.137

Figures in bold denote statistical significance. * Cox regression analysis. AdjHR = adjusted hazard ratio; CI = confidence interval.
